# Risk and mortality of aspiration pneumonia in Parkinson’s disease: a nationwide database study

**DOI:** 10.1038/s41598-021-86011-w

**Published:** 2021-03-23

**Authors:** Jun Hee Won, Seong Jun Byun, Byung-Mo Oh, Sang Jun Park, Han Gil Seo

**Affiliations:** 1 Department of Rehabilitation Medicine, Seoul National University College of Medicine, Seoul National University Hospital, 101 Daehak-ro, Jongno-gu, Seoul, 03080 Republic of Korea; 2 Department of Ophthalmology, Seoul National University College of Medicine, Seoul National University Bundang Hospital, 82, Gumi-ro 173 Beon-gil, Bundang-gu, Seongnam-si, 13620 Gyeonggi-do Republic of Korea; 3 Big Data Center, Seoul National University Bundang Hospital, Seongnam, Republic of Korea

**Keywords:** Health care, Neurology, Risk factors

## Abstract

This retrospective cohort study investigated the risk and mortality rate due to aspiration pneumonia in patients with Parkinson’s disease (PD) using a nationwide database. We identified 10,159 newly diagnosed PD patients between 2004 and 2006, and four age- and sex-matched controls for each PD patient from the National Health Insurance Service database in Korea. We analyzed the relative risk of aspiration pneumonia and mortality after the first occurrence of aspiration pneumonia until 2017. Throughout the study period, PD patients showed a higher incidence of aspiration pneumonia than their matched controls (3.01 vs. 0.59 events per 1,000 person-years), and they were at an increased risk of aspiration pneumonia (hazard ratio = 4.21; 95% confidence interval, 3.87–4.58). After the first occurrence of aspiration pneumonia, the mortality rate of PD patients was 23.9% after one month, 65.2% after 1 year, and 91.8% after 5 years, while that of controls was 30.9%, 67.4%, and 88.9%, respectively. Patients with PD are at an increased risk of aspiration pneumonia, and approximately two-thirds of the patients die within a year after experiencing aspiration pneumonia. Further studies are warranted to prevent aspiration pneumonia and implement proper treatments to prevent death after aspiration pneumonia in patients with PD.

## Introduction

Aspiration pneumonia is a major burden in patients with Parkinson’s disease (PD). It accounts for 70% of deaths among patients with PD^1^, and its incidence in PD patients has rapidly increased recently^2^. Numerous studies have reported that PD is an important risk factor for aspiration pneumonia because it leads to oropharyngeal dysphagia^3–5^. However, few studies have investigated the incidence or frequency of aspiration pneumonia in patients with PD^2,6^. Although a previous study reported a relatively higher incidence of aspiration pneumonia among PD patients^2^, the risk of aspiration pneumonia due to PD has not been investigated. Additionally, because the study was based on a hospital discharge database, it did not report the incidence of aspiration pneumonia among the community-dwelling PD population. Furthermore, precise epidemiologic data on aspiration pneumonia in patients with PD, including annual incidence from the diagnosis of PD and long-term mortality, are scarce.

The South Korean government provides universal insurance coverage through a mandatory health insurance program, named the National Health Insurance Service (NHIS)^7^. As the NHIS database contains information on all healthcare services used by the entire Korean population (approximately 50 million people), it can provide reliable data of PD patients and the general population. Thus, we investigated the relative risk and epidemiology of aspiration pneumonia in patients with PD using this nationwide database.

## Methods

### Data source

We acquired patient information and healthcare utilization data from the NHIS database in South Korea for the 2002–2017 period. As the NHIS program covers 97% of residents in South Korea, the NHIS database contains information regarding healthcare utilization, such as demographic characteristics, diagnosis, procedures, and drug utilization records^8^. In addition, mortality data were obtained from Statistics Korea and merged with the NHIS data.

The NHIS database records diagnoses according to the International Classification of Diseases, 10th edition (ICD-10). The database also contains data from the registration program for rare, intractable diseases (V-code)^9^. This registration program was implemented by the Korean government to reduce the healthcare costs incurred by patients with rare or intractable diseases in 2001. PD was included in this program in 2004^10^. To enroll patients with PD in this program, physicians should check the following criteria: (1) presence of bradykinesia (a minimum score of 2 in the bradykinesia items of the Unified Parkinson’s Disease Rating Scale) with at least one of the following features: muscle rigidity, rest tremor, and/or postural instability; (2) absence of secondary Parkinsonism symptoms, such as stroke, head injury, encephalitis, hypoxic brain injury, and adverse effects of medication; and (3) presence of three or more of the following features: unilateral onset of the symptoms, rest tremor, progressive disorder, persistent asymmetry primarily affecting the side of onset, an excellent response (70–100%) to levodopa, severe levodopa-induced chorea, levodopa response for five years or more, and/or a clinical course for 10 years or more. Patients who met criteria (1) and (2) were registered in this program, and patients who met all three criteria were diagnosed with PD. Even though the requirements are similar to the UK brain bank criteria for the diagnosis of PD^11^, this registration criterion does not definitely exclude atypical parkinsonism, such as multiple system atrophy, progressive supranuclear palsy, and corticobasal syndrome.

### Identifying newly diagnosed PD patients and matched controls for each PD patient

The study population comprised newly diagnosed patients with PD and their matched controls. First, we identified PD patients using the registration code for PD (V124) in the program for rare, intractable disease from January 1, 2004, to December 31, 2006, and we defined the index date as the date of the earliest claim with the V124 code. To remove any prevalent cases, we excluded patients who had PD diagnostic codes (G20) or PD registration codes (V124) before January 1, 2004. As the V124 registration criteria did not exclude atypical Parkinsonian syndromes, we excluded patients diagnosed with atypical parkinsonism (G21, G22, and G23) during the entire study period, from 2002 to 2017. Moreover, we excluded patients under 40 years of age. Lastly, we excluded patients whose total number of days of antiparkinsonian medications was less than 180 days. The list of the antiparkinsonian medications used in this study is given in Supplementary Table S1.

Then, we selected up to four controls for each PD patient matched by sex and age at the index date. Previous studies reported that matching 4 controls to 1 patient can minimize the bias in measuring treatment effect in the maximum number of matched controls^12,13^. Individuals who had the registration code for rare, intractable disease for PD (V124), had any diagnostic code for Parkinsonism (G20, G21, G22, and G23), or had been prescribed an antiparkinsonian drug during the study period (2002–2017) were not recruited as controls.

### Definition of demographics and confounders

We defined age, sex, residential region, and household income in reference to the index date. We also defined the presence of comorbidities according to previous diagnoses up to two years before the index date. The defined comorbidities included diabetes mellitus, hypertension, ischemic heart disease, congestive heart failure, cancer, tuberculosis, peripheral arterial disease, atrial fibrillation, chronic kidney disease, dyslipidemia, cerebrovascular disease, dementia, chronic obstructive pulmonary disease, and seizure disorder^3,14^. Information on medications, including anticoagulants, antihypertensive agents, oral hypoglycemic agents, insulin, benzodiazepines, and antipsychotics was collected from the prescription records within two years from the index date. The list of co-medications is provided in Supplementary Table S2. Modified Charlson comorbidity index scores were calculated from the previous diagnosis within a year before the index date. These diagnoses include diagnoses of myocardial infection, congestive heart failure, peripheral vascular disease, cerebrovascular disease, dementia, chronic pulmonary disease, rheumatologic disease, peptic ulcer disease, diabetes without chronic complications, diabetes with chronic complications, hemiplegia, renal disease, any malignancy including leukemia and lymphoma, mild liver disease, moderate or severe liver disease, metastatic solid tumor, and AIDS^15^.

### Statistical analysis

The primary outcome was the development of aspiration pneumonia; the incidence of aspiration pneumonia was identified by the relevant diagnostic codes (J69) for hospital admission from the index date to the end of the study period (December 31, 2017).

First, we assessed the incidence rates of aspiration pneumonia in both PD patients and their controls. As aspiration pneumonia could occur repeatedly, we distinguished between the occurrence of new aspiration pneumonia and its persistence by applying a 60-day washout period. If an episode of aspiration pneumonia occurred 60 days after the last episode, it was regarded as a new event; however, if it occurred within 60 days, it was regarded as a persistent previous event. We then calculated the annual incidence of aspiration pneumonia from the index date.

Second, to compare aspiration pneumonia-free survival between patients with PD and the control group, we plotted Kaplan–Meier curves. In addition, we applied the Cox proportional hazard model to investigate the risk of aspiration pneumonia; we considered demographics, comorbidities, co-medications, and the Charlson comorbidity index score as confounders^16^. In addition, we applied a competing risk model to assess the relative risk of aspiration pneumonia in PD patients^17^, as the occurrence of aspiration pneumonia and death might be competing events. We used the subdistribution hazard model, which provides estimates of risk in subjects who have not yet experienced either aspiration pneumonia or death, or in subjects who died^18^.

Lastly, we used the life table method to assess mortality rates after the first occurrence of aspiration pneumonia in patients with PD and the control group.

We used SAS version 9.4 (SAS Inc., Cary, NC, USA), and STATA software, version 15.0 (Stata Corporation, College Station, TX, USA), for statistical analyses.

### Standard protocol approval, registration, and patient consent

The study protocol was assessed and determined to be exempt from review by the Institutional Review Board of Seoul National University Hospital (E-1810-033-977). Furthermore, the NHIS approved the use of its database and provided data after excluding all possible patient identification information (NHIS-2019-1-084). The requirement for informed consent was waived by the Institutional Review Board of the Seoul National University, because the database was anonymized. All methods were carried out in accordance with relevant guidelines and regulations.

## Results

We identified 10,159 patients (6,272 women, 61.7%) with incident PD and 39,574 matched controls (24,508 women, 61.9%); therefore, a total of 49,733 individuals were included in the analyses. A flowchart for selecting patients with PD is presented in Supplementary Figure S1. The prevalence of dementia was 12.8% and 2.3% in PD patients and control subjects, respectively. Table 1 shows detailed information regarding the characteristics of the study population.

**Table 1:** Baseline characteristics of study population and control group.

Population		Patients with PD (N = 10,159)		Patients without PD (N = 39,574)		P-value
Characteristics		No	%	No	%	
Sex	Male	3887	38.3	15,066	38.1	
	Female	6272	61.7	24,508	61.9	
Age at diagnosis (year)	40–50	319	3.1	1274	3.2	
	50–60	923	9.1	3670	9.3	
	60–70	2943	29.0	11,644	29.4	
	70–80	4487	44.2	17,434	44.1	
	80–	1487	14.6	5552	14.0	
Region of residence	Seoul and Incheon	2202	21.7	8940	22.6	< .001
	Gyeonggi and Gangwon	2124	20.9	8734	22.1	
	Busan, Daegu, Ulsan, and Gyeongsang	3267	32.2	11,052	27.9	
	Daejeon, Sejong, and Chungcheong	982	9.7	4727	11.9	
	Gwangju, Jeola, and Jeju	1584	15.6	6086	15.4	
	Not identified	0	–	35	0.1	
Comorbidities	Hypertension	6348	62.5	17,971	45.4	< .001
	Diabetes	3822	37.6	9279	23.4	< .001
	Ischemic heart disease	2297	22.6	5263	13.3	< .001
	Congestive heart failure	1268	12.5	2905	7.3	< .001
	Cancer	901	8.9	2474	6.3	< .001
	Tuberculosis	379	3.7	1096	2.8	< .001
	Peripheral arterial disease	1840	18.1	3924	9.9	< .001
	Atrial fibrillation	414	4.1	845	2.1	< .001
	Chronic kidney disease	187	1.8	323	0.8	< .001
	Dyslipidemia	3350	33.0	8080	20.4	< .001
	Cerebrovascular disease	3605	35.5	3992	10.1	< .001
	Dementia	1301	12.8	907	2.3	< .001
	COPD	3640	35.8	12,708	32.1	< .001
	Seizure disorder	974	9.6	607	1.5	< .001

*PD* Parkinson’s disease, *COPD* chronic obstructive pulmonary disease.

Charlson comorbidity index was included in the analysis.

The incidence of aspiration pneumonia was higher in PD patients than in the matched controls; the mean incidence was 3.01 events/1000 person-years in the PD patients and 0.59 events/1000 person-years in the controls. Table 2 presents detailed information on the annual incidence rates in each group after the index date.

**Table 2:** Incidence of aspiration pneumonia among PD patients and control groups in each year.

Follow up Duration (year)	Incidence (events per person-year)		Incidence (person per person-year)	
	Patients with PD	Patients without PD	Patients with PD	Patients without PD
≤ 1	2.646	0.362	1.508	0.223
> 1, ≤ 2	3.479	0.425	1.874	0.262
> 2, ≤ 3	3.302	0.513	1.684	0.292
> 3, ≤ 4	3.481	0.442	1.553	0.223
> 4, ≤ 5	2.495	0.602	1.447	0.264
> 5, ≤ 6	2.869	0.576	1.248	0.270
> 6, ≤ 7	3.039	0.546	1.384	0.298
> 7, ≤ 8	2.704	0.629	1.344	0.356
> 8, ≤ 9	2.990	0.565	1.411	0.328
> 9, ≤ 10	2.949	0.748	1.403	0.371
> 10, ≤ 11	3.341	0.930	1.410	0.418

*PD* Parkinson’s disease.

**Figure 1:**
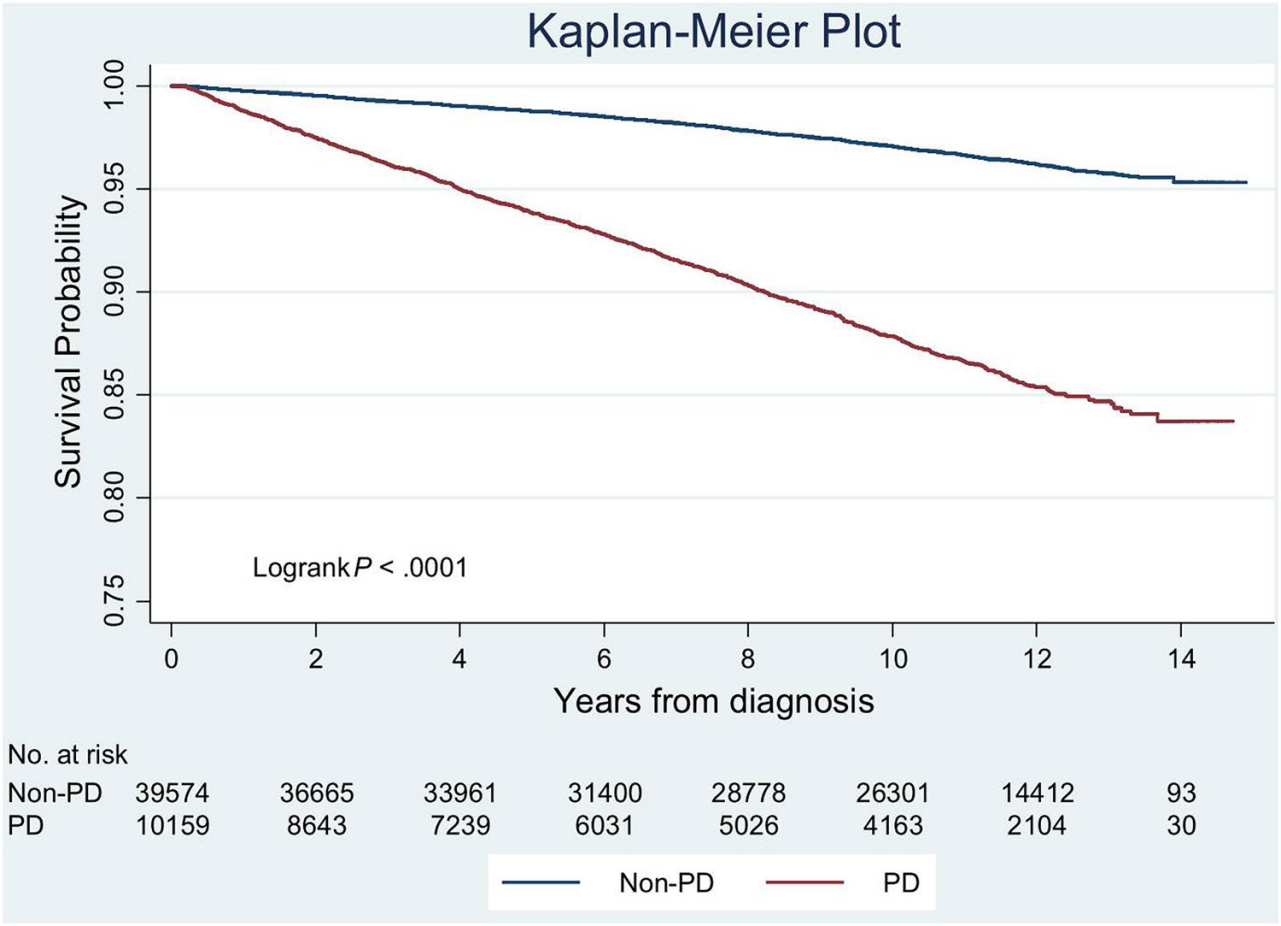
Kaplan Meier analysis for first occurrence of aspiration pneumonia in PD patients and control group.

The Kaplan–Meier plot showed that PD patients were at a higher risk of aspiration pneumonia than the control group individuals (p < 0.0001, log-rank test; Fig. 1). After adjusting for confounders, the Cox proportional hazards model showed a significantly higher risk of aspiration pneumonia in PD patients (adjusted hazard ratio = 4.21; 95% confidence interval [CI], 3.87–4.58). Additionally, it also showed that old age, male sex, tuberculosis, cerebrovascular disease, dementia, and usage of antidiabetic drugs, anticoagulants, and antiplatelet drugs were associated with a higher risk of aspiration pneumonia. In contrast, peripheral artery disease was associated with a lower risk of aspiration pneumonia (Table 3). Analysis using the subdistribution hazards model, in which death was treated as a competing risk of aspiration pneumonia, also revealed a significantly higher risk of aspiration pneumonia in patients with PD (subdistribution hazard ratio = 3.33; 95% CI, 3.06–3.62).

**Table 3:** Cox proportional hazard model for risk factors of aspiration pneumonia.

		Adjusted HR	(95% CI)	P-value
PD*		4.21	3.87–4.58	< .0001
Sex	Male	Ref	–	
	Female	0.416	0.382–0.454	< .0001
Age at diagnosis (year)	40–50	Ref	–	
	50–60	2.45	1.299–4.623	0.0057
	60–70	6.391	3.513–11.626	< .0001
	70–80	12.985	7.155–23.566	< .0001
	80 +	22.253	12.196–40.603	< .0001
Comorbidities	Hypertension	0.971	0.865–1.089	0.6127
	Diabetes	1.1	0.983–1.232	0.0973
	Ischemic heart disease	1.022	0.909–1.15	0.7144
	Congestive heart failure	1.121	0.971–1.294	0.1197
	Cancer	0.959	0.811–1.134	0.6275
	Tuberculosis	1.429	1.17–1.744	0.0005
	Peripheral arterial disease	0.849	0.748–0.963	0.0107
	Atrial fibrillation	1.127	0.887–1.431	0.3278
	Chronic kidney disease	1.265	0.903–1.772	0.1712
	Dyslipidemia	0.909	0.817–1.01	0.0767
	Cerebrovascular disease	1.322	1.181–1.479	< .0001
	Dementia	1.858	1.607–2.149	< .0001
	COPD	0.994	0.902–1.094	0.8952
	Seizure disorder	0.992	0.827–1.19	0.9292
Comedications	Antihypertensive drug	0.993	0.881–1.119	0.9053
	Antidiabetic drug	1.249	1.073–1.454	0.0041
	Anticoagulant	1.68	1.102–2.56	0.0158
	Antiplatelet drug	1.206	1.048–1.387	0.0089
	Benzodiazepines, antipsychotics	0.951	0.821–1.103	0.5099

*PD* Parkinson’s disease, *HR* hazard ratio, *CI* confidence interval, *COPD* chronic obstructive pulmonary disease.

*Adjusted for age group, sex, region of residence, the Charlson comorbidity index score, comedications and comorbidities.

Lastly, the mortality rates of patients with PD after the first occurrence of aspiration pneumonia were 23.9% after 1 month, 65.2% after 1 year, and 91.8% after 5 years, while those of controls were 30.9%, 67.4%, and 88.9%, respectively (Table 4).

**Table 4:** Mortality after occurrence of aspiration pneumonia among PD patients and control group.

Follow up duration	Mortality rate (percent)	
	Patients with PD (%)	Patients without PD (%)
1 month	23.9	30.9
3 months	41.8	48.0
6 months	54.1	57.0
12 months	65.2	67.4
24 months	76.3	76.8
36 months	84.4	81.8
48 months	88.3	86.1
60 months	91.8	88.9

*PD* Parkinson’s disease.

## Discussion

The present study found a higher incidence and risk of aspiration pneumonia in patients with PD than in non-PD controls. Moreover, this study revealed that approximately two-thirds of patients died within a year after the first occurrence of aspiration pneumonia. For this study, we identified more than 10,000 newly diagnosed PD patients among the 48 million individuals in the entire country, and we were able to evaluate all individuals for a period of over 10 years without dropping out. To increase the accuracy of identification, we utilized the V124 code for the rare, intractable disease program and prescription claims for antiparkinsonian medication. Thus, our study provides reliable data regarding aspiration pneumonia risk in patients with PD. In the same cohort, we reported that patients with PD had 2.23 times higher risk for pneumonia than the general population^19^. As the hazard ratio is approximately two times higher for aspiration pneumonia, we can suggest that patients with PD are more susceptible to aspiration pneumonia than to pneumonia.

In our study, PD patients had a higher incidence of aspiration pneumonia after diagnosis and throughout the study period. This result may be due to the occurrence of dysphagia, decreased cough reflex, and oral dysfunction. It is well known that dysphagia occurs in the late stages of PD, and it has been reported to increase in severity every year after PD diagnosis^20^. Recently, it has been reported that dysphagia and decreased cough reflex occur in the early stages of PD. Significant pharyngeal dysfunction and aspiration requiring intervention were observed in patients with early stage PD with a disease duration of less than 2 years^21,22^. Furthermore, PD patients showed decreased efficacy of voluntary cough and reflex cough even during the early stages of PD^23^. Moreover, PD patients showed prolonged oral transit time due to lingual bradykinesia.^24^ This could disrupt the subsequent pharyngeal phase, leading to aspiration^25^. Our results suggest that PD patients are vulnerable to aspiration pneumonia even in the early stages. Therefore, early screening and education on the risk of aspiration are essential for patients with PD.

Previous studies have reported the incidence of aspiration pneumonia in hospitalized patients. Akbar et al.^2^ reported a higher incidence of aspiration pneumonia in patients with PD than in non-PD patients (3.8% vs. 1.0%) by investigating 32 years of medical records, while Ramirez et al.^6^ reported that 2.4% of hospitalized PD patients experienced aspiration pneumonia. Because our study population consisted of newly diagnosed community-dwelling PD patients, it is difficult to compare our results with those of previous studies that included hospitalized PD patients regardless of disease stage. However, our results were consistent with those of previous studies that reported a higher incidence of aspiration pneumonia in PD patients than in the general population. Because we evaluated the risk via adjusting comorbidities and matching a control group, we could report more precise data for the risk of aspiration pneumonia.

We observed that most patients who had experienced aspiration pneumonia died within two years, regardless of the presence of PD. This might be due to underlying morbidity and functional decline after aspiration pneumonia. Aspiration pneumonia was reported, in previous studies, to have a higher mortality than other forms of pneumonia^26^. Lanspa et al.^27^ reported that the 30-day mortality rate of aspiration pneumonia was 21%. Yoon et al.^28^ reported that the 1-, 3-, and 5-year mortality rates of aspiration pneumonia were 49.0%, 67.1%, and 76.9%, respectively. They suggested that the poor long-term prognosis of aspiration pneumonia resulted from underlying morbidity. Our study revealed an even higher long-term mortality rate of aspiration pneumonia in both PD patients and controls. Because previous studies investigated patients in tertiary-care centers, the difference in study populations between studies may be a reason for the higher mortality rate in our study. The mortality rate for aspiration pneumonia was lower in patients with PD than in controls for up to 24 months, especially at 1 month. The controls who experienced aspiration pneumonia might have had other comorbidities, such as stroke, that could have caused aspiration pneumonia. These comorbidities could have contributed to the higher initial 1-month mortality in the controls after the occurrence of aspiration pneumonia. However, the higher mortality rate in PD patients than in controls after 36 months may be due to disease progression in PD patients.

Early detection and treatment of dysphagia may lower the initial mortality from aspiration pneumonia in patients with PD. A meta-analysis conducted by van Hooren et al.^29^ revealed that two swallowing therapies, namely, expiratory muscle strength training and video-assisted swallowing therapies, were effective for treating dysphagia in patients with PD. In addition, compensation techniques, including chin-down posture and thickened fluid, can be useful methods for preventing aspiration in patients with PD^30^. Because patients with aspiration pneumonia have a higher risk of recurrent pneumonia^31^, and the long-term mortality rate of aspiration pneumonia is very high, it is crucial to manage dysphagia properly to prevent recurrent aspiration pneumonia in patients with PD.

The risk factors associated with aspiration pneumonia identified in this study were consistent with those reported in previous studies^3,14^. Age was the strongest risk factor for aspiration pneumonia. People who were over 80 years old showed a 22-fold higher risk for aspiration pneumonia than those in their 40 s. Dementia was also associated with an increased risk of pneumonia in this study and any-type dementia is a well-known risk factor for aspiration pneumonia^3^. Patients with Lewy body dementia share similar motor symptoms with PD patients, but have a higher risk of aspiration pneumonia than those with idiopathic PD^32^. In our PD population, the presence of Lewy body dementia might contribute to the higher incidence of aspiration pneumonia. Peripheral artery disease was the only factor associated with a lower risk of aspiration pneumonia in our analysis. To the best of our knowledge, no study has investigated the association between peripheral artery disease and aspiration pneumonia. Further studies are needed to verify this association.

This study has some limitations. Because we identified aspiration pneumonia according to the ICD-10 code in this study, there is a possibility of inaccurate coding for aspiration pneumonia. However, the identification of pneumonia using the ICD-10 code has been validated in a previous study^33^, and several studies have reported the incidence of aspiration pneumonia using ICD-10 codes^2,27,34^. Similarly, the identification of patients with PD could be inaccurate. To compensate for this, we utilized data from the program for rare, intractable diseases. The Health Insurance Review and Assessment service inspects the accuracy of diagnosis by reviewing the medical records of every patient registered in this program. We also excluded patients according to the prescription records of antiparkinsonian medication. The reliability of PD diagnosis could be verified based on the procedures. Lastly, the incidence of aspiration pneumonia might be overestimated in patients with PD compared to those in the control group. Physicians might be more likely to diagnose dysphagia and aspiration pneumonia in PD patients because PD is a well-known risk factor for aspiration pneumonia. Although this study has the advantage of representing the whole population across a country, hospital-based studies using more accurate diagnostic criteria are also necessary to clarify the incidence and risk of aspiration pneumonia in PD patients.

This is a nationwide study on the risk and mortality rate of aspiration pneumonia in the entire population of PD patients in a single country. PD patients had a four times higher risk of aspiration pneumonia during the subsequent 10-year period after diagnosis, even after adjusting for other confounders. Furthermore, approximately two-thirds of the patients died within a year after the first occurrence of aspiration pneumonia, regardless of PD diagnosis. Therefore, it is essential to assess the risk of aspiration pneumonia and provide appropriate measures to prevent its occurrence during the early stages of PD.

## Supplementary Information

Supplementary Figure S1.

Supplementary Tables.

## Data Availability

All data used for the present study can be obtained through formal application to the National Health Insurance Sharing Service (https://nhiss.nhis.or.kr/bd/ab/bdaba000eng.do).
