# Supplementary material for: Risk and mortality of aspiration pneumonia in Parkinson’s disease: a nationwide database study
Source: Sci Rep. 2021 Mar 23;11:6597. doi: 10.1038/s41598-021-86011-w (PMC7988066; doi:10.1038/s41598-021-86011-w)
Supplement: Supplementary file 2 — Supplementary Tables. [file 41598_2021_86011_MOESM2_ESM.docx]

**Risk and Mortality of Aspiration Pneumonia in Parkinson’s Disease: A Nationwide Database Study**

Jun Hee Won MD^1^, Seong Jun Byun MSc^2^, Byung-Mo Oh MD, PhD^1^, Sang Jun Park MD, MSc^2, 3*^, Han Gil Seo MD, PhD^1*^

**Supplementary information**

Supplementary Table S1. List of antiparkinsonian medications mentioned in the present study Supplementary Table S2. List of comedications used in the present study

Supplementary Figure S1. Flow chart for the selection of PD patients

Supplementary Table S1. List of antiparkinsonian medications mentioned in the present study.

| Classification | Medication | Generic code |
| --- | --- | --- |
| Levodopa | Levodopa | 183001ATB, 256000ACH, 256000ATB, 256000ATD, 256000ATR, 256100ATR, 256200ATB, 256300ATB, 256400ATB |
|  | Controlled release levodopa | 255900ACR, 256000ACR, 256500ATR |
|  | Duodopa | 639400ACM |
| COMT inhibitors | Entacapone | 439201ATB |
|  | Levodopa-Entacapone | 468300ATB, 468400ATB, 468900ATB, 499900ATB, 507200ATB, 507300ATB |
| Dopamine agonists | Pramipexole | 402501ATB, 402502ATB, 402503ATB, 402504ATB, 402505ATB, 402505ATR, 402506ATR, 402507ATR |
|  | Ropinirole | 224901ATB, 224902ATB, 224903ATB, 224904ATB, 224902ATR, 224905ATR, 224906ATR |
|  | Rotigotine | 612107CPC, 612108CPC, 612109CPC, 612110CPC, 612111CPC, 612112CPC |
|  | Piribedil | 213901ATB |
|  | Lisuride | 184601ATB |
|  | Bromocriptine | 118701ATB |
|  | Pergolide | 211201ATB, 211202ATB, 211203ATB |
| MAOB inhibitors | Selegiline | 226401ATB |
|  | Rasagiline | 625201ATB |
| Other | Amantadine | 106101ACH, 106201ATB |

COMT, catechol-*O*-methyltransferase; MAOB, monoamine oxidase type B

Supplementary Table S2. List of comedications used in the present study

| Classification | Medication | Generic code |
| --- | --- | --- |
| Antiplatelet | Aspirin | 110701ATB, 110702ATB, 110801ATB,  110802ATB, 256800ATB, 517900ACH, 111001ACE, 111001ATB, 111001ATE, 111002ATE, 111003ACE, 111003ATE, 110902BIJ |
|  | Ticlopidine | 498900ATB, 239201ATB, 239202ATB |
|  | Clopidogrel | 517900ACH |
|  | Sulodexide | 233303ACS |
|  | Beraprost | 116201ATB |
|  | Cilostazol | 133201ACR, 133201ATB, 133202ATB,  133203ATR, 506100ATB |
|  | Ginko | 165301ACS, 165301ATB, 165303ACS,  165303ALQ, 165303ATB, 165304ATB, 165305ALQ, 165305ATB |
|  | Indobufen | 174701ATB |
|  | Sarpogrelate | 226101ATB |
|  | Triflusal | 244101ACE, 244101ACH, 244102ACH |
|  | Ibudilast | 172701ACH |
|  | Dipyridamole | 147201ATB |
|  | Abciximab | 100401BIJ, 100402BIJ |
|  | Turifubab | 240201BIJ |
| Anticoagulation | Warfarin | 249103ATB, 249105ATB |
|  | Heparin | 168601BIJ, 168602BIJ |
|  | Dalteparin | 140201BIJ, 140202BIJ, 140203BIJ |
|  | Argatroban | 359601BIJ, 359602BIJ, 359603BIJ |
|  | Enoxaparin | 152101BIJ, 152102BIJ, 152103BIJ,  152104BIJ, 152105BIJ, 152106BIJ |
|  | Fondaprinux | 450101BIJ |
|  | Nadroparin | 198401BIJ, 198402BIJ, 198403BIJ,  198407BIJ |
|  | Dabigatran | 613701ACH, 613702ACH |
|  | Rivaroxaban | 511401ATB, 511402ATB, 511403ATB, 511404ATB |
|  | Apixaban | 617001ATB, 617002ATB |
| Antihypertensive | Alacepril | 104201ATB, 104202ATB |
|  | Benazepril | 114701ATB |
|  | Captopril | 122901ATB, 122902ATB, 122903ATB |
|  | Cilazapril | 133001ATB, 133002ATB, 133003ATB |
|  | Enalapril | 151601ATB, 151603ATB |
|  | Fosinopril | 163501ATB, 163502ATB |
|  | Imidapril | 173401ATB, 173402ATB |
|  | Moexipril | 196801ATB, 196802ATB |
|  | Perindopril | 211301ATB, 211302ATB, 501601ATB, 501602ATB |
|  | Quinapril | 221901ATB |
|  | Ramipril | 222401ATB, 222402ATB, 222404ATB |
|  | Temocapril | 235002ATB |
|  | Zofenopril | 510401ATB, 510402ATB, 510403ATB |
|  | Felodipine/Ramipril | 447100ATB, 447200ATB |
|  | Enalapril/Nitrendipine | 466000ATB |
|  | Candesartan | 122601ATB, 122602ATB, 122603ATB |
|  | Irbesartan | 177301ATB, 177303ATB |
|  | Losartan | 185701ATB, 185702ATB |
|  | Valsartan | 247101ATB, 247102ATB, 247103ATB, 247104ATB |
|  | Telmisartan | 378801ATB, 378802ATB |
|  | Eprosartan | 429201ATB |
|  | Olmesartan | 468501ATB, 468502ATB, 468503ATB, 520901ATB, 520902ATB |
|  | Fimasartan | 515201ATB, 515202ATB, 515203ATB |
|  | Amlodipine/Valsartan | 492800ATB, 492900ATB, 495800ATB, 522900ATB, 523000ATB, 523100ATB, 523200ATB, 523300ATB, 523400ATB |
|  | Amlodipine/Olmesartan | 500500ATB, 500600ATB, 582200ATB, 582400ATB |
|  | Amlodipine/Losartan | 503000ATB, 513900ATB, 502700ATB |
|  | Amlodipine/Telmisartan | 511500ATB, 511600ATB, 511700ATB |
|  | S-amlodipine/Telmisartan | 521200ATB, 521300ATB, 521400ATB |
|  | Lercanidipine/Valsartan | 522200ATB, 522300ATB, 522400ATB |
|  | S-amlodipine/Valsartan | 522600ATB, 522700ATB, 522800ATB |
|  | Fimasartan/Hydrochlorothiazide | 526800ATB |
|  | Amlodipine/Olmesartan/Hydrochlorothiazide | 519700ATB, 519800ATB, 519900ATB, 520000ATB, 520100ATB |
|  | Irbesartan/Atorvastatin | 524000ATB, 524100ATB, 527000ATB, 527100ATB, 527000ATB, 527100ATB |
|  | Valsartan/Rosuvastatin | 525000ATB, 525100ATB, 525200ATB, 525300ATB |
|  | Olmesartan/Rosuvastatin | 526300ATB, 526400ATB, 526500ATB, 526900ATB |
|  | Amlodipine | 107601ATD, 107602ATB, 107602ATD, 107601ATB, 459801ACH, 459801ATB, 459802ACH, 459901ATB, 464601ATB, 470801ATB, 470802ATB, 476201ATB, 479701ATB |
|  | S-amlodipine | 483201ATB, 483202ATB, 486501ATB, 495901ATB |
|  | Felodipine | 157501ATR, 157503ATR |
|  | Isradipine | 178902ACR |
|  | Nicardipine | 201003ACR, 201002ATB, 201001BIJ, 201002BIJ |
|  | Nifedipine | 201401ACS, 201401ATB, 201405ATR, 201407ACS, 201408ATR, 201409ATR |
|  | Nimodipine | 201901ATB, 201902BIJ |
|  | Nisoldipine | 356201ATB, 356202ATB, 356202ATR, 356203ATR |
|  | Lacidipine | 180301ATB, 180302ATB, 180303ATB |
|  | Nilvadipine | 201702ATB |
|  | Manidipine | 188001ATB, 188002ATB |
|  | Barnidipine | 114001ACH, 114002ACH, 114003ACH |
|  | Lercanidipine | 182001ATB, 182002ATB |
|  | Cilnidipine | 133101ATB, 133102ATB |
|  | Benidipine | 115101ATB, 115102ATB, 115103ATB, 115104ATB |
|  | Verapamil | 247603ATR, 247605ATR, 247606ATB, 247607ATB, 247604BIJ |
|  | Diltiazem | 145703ACR, 145706ATB, 145706ATR, 145707ACR, 145707ATB, 145707ATR, 145704BIJ |
|  | Amlodipine/Atorvastatin | 472300ATB, 472400ATB, 472500ATB, 518900ATB |
|  | S-amlodipine/Atorvastatin | 614500ATB |
|  | Felodipien/Metoprolol | 262400ATR |
|  | Timolol | 239601COS, 239602COS, 239607COS |
|  | Sotalol | 230402ATB, 124801ATB, 124802COS, 124806COS, 124807COS |
|  | Carteolol | 124801ATB, 124802COS, 124803COS, 124806COS, 124807COS |
|  | Propranolol | 219901ATB, 219904ATB, 219905ACR, 219906ACR |
|  | Metoprolol | 194003ATR, 193802ATB |
|  | Atenolol | 111402ATB, 111403ATB |
|  | S-atenolol | 483101ATB, 483102ATB |
|  | Bisoprolol | 117903ATB |
|  | Celiprolol | 129101ATB |
|  | Esmolol | 154401BIJ, 154402BIJ |
|  | Nebivolol | 489501ATB, 489502ATB |
|  | Labetalol | 180201BIJ, 180202BIJ |
|  | Carvedilol | 125001ATB, 125003ATB, 125002ATB, 125004ACR, 125005ATB, 125006ACR, 125007ACR, 125008ACR |
|  | Hydrochlorothiazide | 170801ATB |
|  | Metolazone | 367001ATB, 367002ATB |
|  | Xipamide | 249401ATB |
|  | Indapamide | 174401ATR, 174403ATB |
|  | Furosemide | 163801ATB, 163802BIJ |
|  | Torasemide | 242001ATB, 242002ATB, 242003ATB, 242004ATB |
|  | Spironolactone | 231101ATB, 231102ATB |
|  | Amiloride | 106901ATB |
|  | Captopril/Hydrochlorothiazide | 262200ATB, 262300ATB |
|  | Enalapril/Hydrochlorothiazide | 440300ATB, 453700ATB |
|  | Moexipril/Hydrochlorothiazide | 440800ATB, 497900ATB |
|  | Ramipril/Hydrochlorothiazide | 448600ATB, 448700ATB |
|  | Lisinopril/Hydrochlorothiazide | 499200ATB, 499300ATB |
|  | Perindopril/Indapamide | 556200ATB |
|  | Losartan/Hydrochlorothiazide | 262500ATB, 378900ATB, 486900ATB |
|  | Valsartan/Hydrochlorothiazide | 356400ATB, 442600ATB |
|  | Irbesartan/Hydrochlorothiazide | 385700ATB, 385800ATB |
|  | Candesartan/Hydrochlorothiazide | 423700ATB |
|  | Telmisartan/Hydrochlorothiazide | 443200ATB, 502600ATB, 443300ATB |
|  | Eprosartan/Hydrochlorothiazide | 460500ATB |
|  | Olmesartan/Hydrochlorothiazide | 513600ATB |
|  | Fimasartan/Hydrochlorothiazide | 522000ATB, 526800ATB |
|  | Metoprolol/Hydrochlorothiazide | 262600ATB |
|  | Bisoprolol/Hydrochlorothiazide | 469800ATB, 469900ATB, 470000ATB |
|  | Atenolol/Chlorothalidone | 262100ATB, 460200ATB |
|  | Hydrochlorothiazide/Spironolactone | 262700ATB |
| Anti-diabetic | Insulin | 170101BIJ, 170102BIJ, 170401BIJ, 170402BIJ, 175301BIJ, 175302BIJ, 175304BIJ, 441301BIJ, 441302BIJ, 441303BIJ, 441304BIJ, 441305BIJ, 461801BIJ, 461802BIJ, 484901BIJ, 484902BIJ, 488701BIJ, 507401BIJ, 512101BIJ, 512102BIJ, 626601BIJ, 626602BIJ |
|  | Glibenclamide | 165402ATB |
|  | Glipizide | 165801ATB |
|  | Gliclazide | 165602ATB, 165603ATR, 165604ATR |
|  | Glimepiride | 165701ATB, 165702ATB, 165703ATB, 165704ATB |
|  | Repaglinide | 379501ATB, 379502ATB, 379503ATB |
|  | Nateglinide | 430201ATB, 430202ATB, 430203ATB |
|  | Metformin | 191501ATB, 191502ATB, 191502ATR, 191503ATB, 191504ATB, 191504ATR, 191505ATR |
|  | Rosiglitazone | 348002ATB |
|  | Pioglitazone | 431901ATB, 431902ATB |
|  | Gemigliptin | 619101ATB |
|  | Sitagliptin | 501101ATB, 501102ATB, 501103ATB |
|  | Vildagliptin | 500801ATB |
|  | Saxagliptin | 613301ATB, 613302ATB |
|  | Linagliptin | 616401ATB |
|  | Alogliptin | 624201ATB, 624202ATB, 624203ATB |
|  | Acarbose | 100601ATB, 100602ATB |
|  | Miglitol | 406201ATB, 406202ATB |
|  | Voglibose | 249001ATB, 249001ATD, 249002ATB, 249002ATD |
|  | Glimepiride/Rosiglitazone | 488800ATB, 488900ATB, 489000ATB |
|  | Glimepiride/Pioglitazone | 525500ATB, 525600ATB |
|  | Glibenclamide/Metformin | 421100ATB, 443400ATB, 443500ATB, 471900ATB |
|  | Rosiglitazone/Metformin | 452700ATB, 452900ATB, 469100ATB |
|  | Glibenclamide/Metformin | 471900ATB |
|  | Glimepiride/Metformin | 474200ATB, 474300ATB, 474300ATR, 498600ATB |
|  | Gliclazide/Metformin | 497200ATB |
|  | Pioglitazone/Metformin | 498100ATB |
|  | Sitagliptin/Metformin | 502300ATB, 502900ATB, 502300ATR, 524700ATR, 513700ATB, 513700ATR, 524700ATR |
|  | Vildagliptine/Metformin | 507000ATB, 507100ATB, 519600ATB |
|  | Saxagliptin/Metformin | 518500ATR, 518600ATR |
|  | Mitiglinide/Metformin | 518800ATB |
|  | Linagliptin/Metformin | 520500ATB, 520600ATB, 520700ATB |
|  | Voglibose/Metformin | 523600ATB, 523700ATB |
|  | Gemigliptin/Metformin | 523800ATR |
| Benzodiazepines | Diazepam | 142901ATB. 142901BIJ. 142902ATB.  142903ATB. 142903BIJ |
|  | Chlordiazepoxide | 255800ATB, 131201ATB, 131202ATB |
|  | Clobazam | 135701ATB, 135702ATB |
|  | Oxazepam | 385101ATB |
|  | Alprazolam | 105501ATB, 105502ATB, 105503ATB,  105504ATB, 105505ATB, 105505ATR,  105506ATB |
|  | Lorazepam | 185501ATB, 185502BIJ, 185503ATB,  185504ATB, 185505BIJ |
|  | Temazepam | 271900ATB, 372000ATB |
|  | Clonazepam | 136401ATB |
| Antipsychotics | Chlorpromazine | 131901ATB, 131902BIJ, 131903ATB,  131904BIJ, 131905ATB, 131906BIJ,  131907ATB, 131908ATB |
|  | Levomepromazine | 183301ATB, 183302ATB, 183303ATB |
|  | Flupentixol | 161701ATB, 161702ATB, 161703ASY,  161704ATB, 161601BIJ |
|  | Chlorprothixene | 132101ATB, 132102ATB, 132103ATB |
|  | Zuclopenthixol | 134401ATB, 134402ATB |
|  | Haloperidol | 167901ATB, 167902BIJ, 167903ATB,  167904ATB, 167905ATB, 167906ATB,  167907ATB, 167908ATB, 167909ATB  168001BIJ |
|  | Melperone | 189801ATB, 189802ATB |
|  | Levosulpiride | 183501ATB |
|  | Thioridazine | 238001ATB, 238002ATB, 238003ATB,  38004ATB |
|  | Loxapine | 186001ACH, 186002ACH, 186003ACH |
|  | Perphenazine | 211401ATB, 211402BIJ |
|  | Prochlorperazine | 455201CSP, 448501ATB, 448502ATB |
|  | Pimozide | 212401ATB, 212402ATB |
|  | Olanzapine | 204001ATB, 204001ATD, 204001BIJ,  204002ATB, 204002ATD, 204003ATB,  204004ATB, 204005ATB |
|  | Quetiapine | 378601ATB, 378602ATB, 378603ATB,  378605ATR, 378606ATR, 378607ATR,  378608ATR, 378609ATR, 378604ATB |
|  | Risperidone | 224201ALQ, 224201ATB, 224201ATD,  224202ALQ, 224202ATB, 224202ATD,  224203ATB, 224204ALQ, 224204ATB,  224204ATD, 224207ATB, 224205BIJ,  224206BIJ |
|  | Paliperidone | 503201ATR, 503202ATR, 503203ATR |
|  | Aripiprazole | 451501ATB, 451502ATB, 451503ATB,  451504ATB |
|  | Amisulpiride | 420001ATB, 420002ATB, 420002ATB,  420003ATB, 420004ATB, 420002ATB,  420003ATB, 420004ATB |
|  | Ziprasidone | 464901ACH, 464902ACH, 464903ACH,  464904ACH |
|  | Clozapine | 137501ATB, 137502ATB |
